# Supplementary material for: Are carbon emissions trading and green financial instruments synergistic? -Comprehensive quantitative research based on content analysis
Source: PLoS One. 2024 Mar 7;19(3):e0298601. doi: 10.1371/journal.pone.0298601 (PMC10919601; doi:10.1371/journal.pone.0298601)
Supplement: S1 Data — (ZIP) [file pone.0298601.s001.zip › Data/地级市Z_F工作报告.zip!/║╙─╧/╓▄┐┌╩╨/└┤╫╘╣1⁄2╓┌║┼í╛┬φ┐╦╩2╛▌═°í┐_2011.docx]

各位代表：

　　现在，我代表市人民政府，向大会报告政府工作，请予审议，并请各位政协委员和其他列席人士提出意见。

　　一、2010年及“十一五”工作回顾

　　2010年，面对复杂多变的经济环境和社会发展中的诸多困难，在省委、省政府和市委的正确领导下，全市上下坚持以科学发展观为指导，坚持“四个重在”实践要领，抓住“转方式、调结构、抓带动、上水平、增效益、惠民生、保稳定”七个关键，用实干砥砺斗志，靠发展振奋士气，迎难而上，开拓进取，较好地完成了市二届人大五次会议确定的各项目标任务，经济社会保持了平稳较快发展态势。

　　——经济总量迈上新台阶。初步预计，全市生产总值为1185.2亿元，增长11%，其中第一产业增加值为347亿元，增长4%；第二产业增加值为535.6亿元，增长15.9%；第三产业增加值为302.6亿元，增长9.6%。

　　——粮食生产再创新高。粮食总产量为144.7亿斤，较上年增产了1.36亿斤，连续7年丰产增收，总产量居省辖市第一位。

　　——工业经济快速发展。规模以上工业增加值为350亿元，增长20.5%；主营业务收入达1200亿元，增长30%；实现利润150亿元，增长28%；工业投资293.3亿元，增长15%。

　　——财政实力明显增强。财政一般预算收入为38.3亿元，增长24.1%，其中市本级一般预算收入突破10亿元，增长26.6%；财政一般预算支出193.3亿元，增长18.7%。

　　——金融运行态势良好。金融机构各项存款余额930.5亿元，较年初增加138亿元，增长17.4%；各项贷款余额563.2亿元，较年初增加63.4亿元，增长12.7%。

　　——内外贸易繁荣活跃。社会消费品零售总额483.1亿元，增长18.7%。外贸进出口总额3.8亿美元，增长18.4%，其中出口1.8亿美元，增长28.7%。

　　——民生得到持续改善。城镇新增就业人员8.5万人，下岗失业人员再就业4.3万人，城镇登记失业率控制在4%以内。城镇居民人均可支配收入12455元，农民人均纯收入4270元，分别实际增长6.4%和6.5%。就医、就学、住房和社会保障等关系群众切身利益的问题进一步得到缓解。

　　——社会保持和谐稳定。及时化解人民内部矛盾，妥善处置各类突发事件，严厉打击违法犯罪，安全生产形势持续稳定好转，信访工作得到了省委的肯定，消防工作受到了省政府的表彰，平安周口建设成效明显。

　　一年来，我们突出抓了以下重点工作：

　　（一）坚定不移抓好项目建设。坚持以项目建设为抓手，建立健全重大项目联审联批制和领导分包项目责任制，开展重点项目观摩点评活动，优化项目建设环境，拓宽项目融资渠道，确保投资对经济发展的持续拉动。全社会固定资产投资完成794.2亿元，增长15%。中央四批投资项目开工率、地方财政资金配套到位率和国家、省检查中发现问题整改到位率，均实现了国家、省提出的百分之百的目标要求。71个“转型升级双千工程”项目投资86亿元，超额完成年度投资计划25个百分点。全市100个重点项目投资116.3亿元，超额完成年度投资计划10.7个百分点，其中有47个项目已建成投用。“双十五”工程投资68.7亿元，超额完成年度投资计划9.3个百分点，其中的金丹乳酸公司高品质乳酸等10个项目已建成投用。

　　（二）持之以恒培育壮大工业经济。立足周口实际，加强服务扶持，工业经济在化解诸多不利因素中，实现了速度、质量、效益同步提升。全部工业增加值完成470亿元，增长14.4%。工业用电量27.7亿千瓦时，增长16.3%。全力服务企业发展。进一步完善企业服务联席会议、市县领导定点联系帮扶企业等行之有效的做法，切实为企业解难题、办实事、谋出路。加强银企对接。8家金融机构共签约项目206个，涉及金额149.6亿元。推进融资担保体系建设。担保机构总数达到52家，形成了30亿元的担保能力，提供贷款担保14.2亿元，使企业融资难题逐步缓解。加强中小企业服务体系建设。我市被省工信厅批准省级中小企业创业基地3个、省级中小企业公共服务平台4个。不断壮大优势骨干企业。规模以上工业企业较年初增加了116家，达到1086家。主营业务收入亿元以上企业达179家，其中突破50亿元的企业有4家。财鑫、辅仁跻身中国民营企业500强。龙源纸业成为全省最大的A级瓦楞纸生产基地。金丝猴集团等9家企业被省政府认定为“百强民营企业”。莲花天安食业等8家企业被认定为“高新技术企业”。久通电缆等7家企业被认定为“高成长性中小企业”。鑫达纺织等4家企业被评为“河南省纺织服装行业50强企业”。实施品牌带动和质量兴市战略。莲花股份成为全省惟一的“国家商标战略实施示范企业”。长城门业“安利来”、康鑫药业“百年康鑫”、辅仁集团“辅仁”被认定为“中国驰名商标”。我市中国驰名商标达9件，总量居全省第二位。祥龙四五等16种产品被认定为“河南省名牌”。金丹乳酸、地神种业、宋河酒业3家企业获首届“市长质量奖”。

　　（三）坚持不懈做好“三农”工作。认真贯彻落实党的强农惠农政策，农民减负增收16.3亿元。加强农业基础设施建设。筹集资金4.6亿元，新增有效灌溉面积31.8万亩、旱涝保收面积4.7万亩，改造中低产田12.4万亩，建设高标准农田3万亩。新增大中型农业机械3718台，农业机械化水平稳步提升。调整优化种植结构。高效特色农业保护栽培面积达320万亩，无公害农产品生产基地达84个，无公害农产品达21种，绿色食品达28种。加快发展畜牧养殖业。新建188个大中型规模养殖场区，其中有11家规模养殖企业成为“全国首批畜禽规模养殖标准化示范企业”。

全年实现畜牧业总产值169亿元，增长4.2%。积极推进林业生态建设和林产业发展。10个县市区全部通过林业生态县验收，木材年加工能力达到107万立方米。规范发展农民专业合作社。农民专业合作组织达2647家，较上年增加1023家，总数居全省第二位。稳步推进新农村建设。继续实施“111”工程，20个新型农村社区、100个试点村建设初见成效。集中整治99个小村镇，创建9个省级生态文明村、1个省级生态镇，村镇面貌得到明显改观。进一步改善农村生产生活条件。完成7个县农村危房改造任务。改造县乡公路394公里，修建通村公路344公里，改造大中型危桥37座。解决300个村、55.7万人的饮水安全问题。完成1670个“村邮站”建设任务。“万村千乡”市场工程新建改造农家店550家。完成125个扶贫开发重点村整村推进任务，17.2万农村人口实现脱贫。

　　（四）多措并举加强城乡规划建设与管理。加大城镇规划、建设、管理力度，城镇化进程迈上新台阶，城镇化率达到31%。进一步完善城乡规划。编制完成县域村镇体系规划、乡镇总体规划和中心村的村庄规划，为统筹城乡发展提供了科学依据。扎实推进中心城区建设。建成区面积60.8平方公里，人口增加到61万人。拓宽改造周口大道，贯通文昌大道大庆路至中州路段，建成投用大庆路桥，实现老城区、开发区、东新区的快速连接，促进了“三区”一体化协调发展。东新区、开发区和老城区部分次干道建设和亮化工程如期竣工，完善了城市路网，改善了交通状况，方便了市民出行。续建或完成市体育馆、市文化艺术中心、市气象服务中心和周口科技职业学院、周口卫校、周口幼师等项目，强化了城市功能，提升了城市品位。建成投用东新区110千伏输变电工程、城市供水应急工程、5处低压区供水管网改造工程和10座公厕、5座垃圾中转站等城市基础设施，改善了人居环境，提高了市民生活质量。加快县城和重点镇建设。9个县市投入公用设施建设资金14.1亿元，新增城镇道路238公里、供排水管道257公里。新增公共绿地144公顷，全市城市绿地达到1261公顷，绿地率32.2%。加强城市管理。从理顺城市管理体制入手，建立城市管理长效机制。开展百日综合整治活动，着力解决城市违章搭建、占道经营、乱倒垃圾、交通秩序混乱等难点问题，市容市貌明显改善。

　　（五）着眼长远加快转变经济发展方式。大力推进结构调整和产业升级，着力提升发展质量和效益。加快产业集聚区建设。11个产业集聚区累计投资585亿元，建成区面积61.4平方公里，入驻企业662家，完成销售收入590.4亿元，实现入库税收20.4亿元。特色产业优势逐步彰显。沈丘聚酯网特色产业园成为全国最大的聚酯网生产、销售和研发基地。太康锅炉产业省内市场占有率达48%以上。不断提升自主创新能力。鼓励和引导企业加快创新平台建设，新建市级工程技术研究中心18家、省级工程技术研究中心5家。累计投入资金35亿元，实施科信电缆碳纤维导线等60个技术改造项目，远东生物“除草剂安全添加剂”被认定为国家重点新产品，科信电缆、瑞特电气2家企业被列入国家科技型中小企业创新基金项目。市经济开发区被评为“河南省创新型产业集聚区”。沈丘秸秆生物质能源开发利用被省政府评为发展成果奖。全市专利授权量增长15%。积极促进企业战略重组。全市26家企业实施联合重组，引入人民币20.8亿元、美元2938.4万元，盘活资产23.7亿元，安置就业2万多人，新增销售收入68.2亿元、利润6.3亿元。鼓励、支持、引导非公有制经济发展。全市新增私营企业2405户、个体工商户2.8万户。大力发展服务业。在巩固提升传统服务业的同时，加快发展现代服务业。书画古玩经营年创收突破1亿元，汝阳刘笔业被文化部命名为“国家文化产业示范基地”。淮阳跻身全国首批5个旅游标准化试点县，淮阳龙湖被国家林业局命名为国家湿地公园；吉鸿昌将军纪念馆通过国家3A级景区评定。周口黄淮物流农产品批发大市场一期工程建成投用。房地产开发投资115亿元，增长20%。保险行业保费收入60亿元，增长44.2%。加强节能减排和生态环境建设。淘汰落后产能企业20家，规模以上工业单位增加值能耗下降14%。6个河流断面化学需氧量、氨氮平均达标率分别达到省政府目标要求，节能减排完成“十一五”目标任务。

　　（六）积极稳妥推进改革开放。把深化改革、扩大开放作为应对复杂局面的综合性、全局性举措，以改革破难题，以开放带全局。扎实推进重点领域改革。政府机构改革全面完成。国企改革继续深化，16家市属国有及国有参股企业改制工作有序推进。医药卫生体制、文化体制改革加快推进，警务体制改革取得突破。农村土地流转服务平台建设步伐加快，全市规范化土地流转面积108万亩，较上年增加15.7万亩。积极推进集体林权制度改革，确权率达到99.5%。深入开展大招商活动。先后在广东东莞、深圳，浙江台州和台湾地区多次举办行业招商活动，投资16亿元的沈丘上海产业园区等一批亿元以上项目落户我市。全市实际利用省外资金178亿元，增长17.2%；实际利用外资1.6亿美元，增长39%。我市被省政府表彰为“对外开放工作先进市”。主动承接产业转移。抢抓台资制鞋企业向内地转移机遇，出台优惠政策，吸引凯鸿鞋业、强鸿鞋业等12家制鞋企业陆续落户，我市被省政府命名为“河南省制鞋产业出口基地”。同时，沈丘凯旺电子、项城鹏宸制衣、市经济开发区集立鞋业等一批劳动密集型产业转移项目开工建设和生产。全市共承接产业转移项目145个，到位资金45.5亿元。

　　（七）尽心尽力保障改善民生。用于保障改善民生的财政支出97亿元，占财政总支出的50.2%。“十项民生工程”取得明显成效。积极做好就业再就业工作。实施“万人创业计划”，落实创业引导资金1890万元，发放小额担保贷款1.6亿多元，带动12.3万人就业。实施“农村劳动力技能就业计划”，新增农村劳动力转移就业15.7万人。不断完善社会保障体系。养老保险、失业保险、医疗保险、工伤保险和生育保险参保人数持续增加，养老金、失业金发放率达100%，社会保障能力不断提升。企业离退休人员养老金人均月增147.9元，城乡低保补助水平、农村“五保”对象供养标准进一步提高。商水被省政府表彰为“社会化养老服务示范县”。扶沟、项城新农保试点工作取得较好成效。农村居民新农合参合率为97.4%。城镇居民医保参保率达到83%。新增保障性住房36.5万平方米，改造棚户区388户、3.5万平方米，困难群体住房难问题得到缓解。加强价格管理和调控，对低收入群体进行补贴，保障了市场物价基本稳定和群众基本生活。大力发展慈善事业和残疾人事业，募集慈善资金1066万元，免费对6236名残疾人进行了职业技能培训。全面发展社会事业。编制了教育发展规划。完成农村中小学校舍维修改造18.2万平方米，“两免一补”资金全部落实；高招工作再创佳绩，本科以上上线率较上年增长20.1个百分点；强力推进职业教育攻坚，2所学校申报为国家级重点职业学校，周口职业教育园区被省政府确定为省重点建设示范性职业教育园区。积极开展全民健身活动，竞技体育水平不断提升，沈丘籍运动员张志磊在第十六届广州亚运会上，获得男子拳击91公斤以上级冠军。繁荣文化艺术事业，建成27个乡镇综合文化站、2805个文化信息资源共享工程村级基层点，823个村建立了农家书屋，为农村公益放映电影5.9万场。“周末一元剧场”荣获国家社会文化活动政府最高奖“群星奖”；实施精品战略，越调剧《老子》荣获我国舞台艺术最高奖“文华奖”，电影《胡辣汤》、《太昊陵》在全国公开放映。中国作家协会把我市确定为文学创作基地。医疗卫生服务体系逐步健全，建成投用市中医院门诊病房综合大楼，改扩建5个县级医院、12个中心乡镇卫生院和4个城市社区卫生服务中心，完成1003个标准化村卫生室建设。完善突发公共卫生事件应急体系，手足口病防治圆满完成省政府提出的“两高一低”目标任务，甲型H1N1流感防控取得阶段性成效。为3.4万名贫困白内障患者免费实施复明手术，我市被授予“全国白内障无障碍市”荣誉称号。坚持保障发展与保护资源并重，土地管理工作取得新成绩。完善计划生育利益导向机制，稳定保持低生育水平。扶沟、项城、鹿邑、淮阳、西华被表彰为“全国计划生育优质服务县”，商水、沈丘、郸城、川汇区被表彰为“全省计划生育优质服务县”。食品药品安全保障水平进一步提高，我市再次被评为“全省食品安全优秀城市”，淮阳、鹿邑、商水、项城荣获“省级食品安全示范县”称号。扎实开展“家电下乡工程”，家电下乡产品销售和财政补贴兑付均居全省前列。

　　（八）深入持久开展精神文明和民主法制建设。广泛开展“迎世博、迎亚运、讲文明、树新风”、“践行文明礼仪我先行”等道德实践活动，43个单位被评为省级文明单位，塑造了文明周口、礼仪周口良好形象。加强文化市场管理，“扫黄打非”斗争取得新成效。自觉接受人大及其常委会法律监督、工作监督和政协民主监督，办理人大代表建议299件、政协提案403件。密切联系工会、共青团、妇联等人民团体，广泛听取社会各界意见和建议。切实加强社会治安综合治理，我市被省综治委表彰为农村平安创建先进市，扶沟荣获“中原平安杯”。深入开展“五五”普法和法律援助工作，法治政府建设扎实推进。加强政府廉政建设，反腐倡廉工作取得新成效。深入开展民族团结进步教育活动，民族宗教工作得到进一步加强。

　　大力开展国防教育，扎实做好双拥工作，进一步巩固军政军民团结，驻周解放军和武警部队在支持地方经济建设、维护社会稳定等方面做出了重要贡献。外事、侨务、对台工作进一步加强，统计、审计、海关、史志、气象、地震、人防等工作取得新成绩。第六次全国人口普查工作进展顺利。圆满完成四川江油大堰乡、西屏乡灾后援建工作。新一轮对口援疆工作开局良好。

　　各位代表，2010年各项工作目标的实现，标志着我市“十一五”主要任务胜利完成。

　　——这五年是综合实力大幅提升的五年。生产总值年均增长在两位数以上，经济总量由595.5亿元增加到1185.2亿元，突破千亿元大关。人均生产总值由691美元增加到1727美元。财政一般预算收入由13.3亿元增加到38.3亿元，其中市本级一般预算收入由3.5亿元增加到10亿元。全部工业增加值由206亿元增加到470亿元。金融机构各项存款余额由500亿元增加到930.5亿元。

　　——这五年是发展后劲显著增强的五年。全社会固定资产投资累计完成2712.9亿元，是“十五”期间的3倍。大广、商周、永登高速公路（周口段）全线通车，新增高速公路通车里程327公里，改善提升农村等级公路5163公里。漯阜铁路（周口段）电气化改造快速推进，沙颍河周口以下航道实现常年通航。中心城区新增日供水能力3.5万立方米，一大批基础设施项目建成投用。

　　——这五年是经济结构进一步优化的五年。三次产业比由34.5∶39.7∶25.8调整为29.3∶45.2∶25.5，二、三产业比重达到70.7%，比“十五”末提高5.2个百分点；非公有制经济占GDP的比重达到61%。

　　——这五年是城乡面貌发生较大变化的五年。城镇建成区面积新增130平方公里，城镇人口新增132万人，是历史上城镇化进程较快的时期。县市区全部建成垃圾和污水处理设施。解决了162.2万人的饮水安全问题，农村广播、电视覆盖率分别达到100%和99.1%，实现了村村通电话、乡乡通宽带，新农村建设迈出坚实步伐。

　　——这五年是人民群众得到实惠更多的五年。用于保障和改善民生的财政支出逐年增长，连续三年占财政总支出的比重超过50%。免费义务教育全面实施，高中阶段教育和高等教育入学率分别达到85%和23%。就业规模持续扩大，累计新增城镇就业人员37万人、农村劳动力转移就业77.3万人。社会事业全面发展，医疗卫生、社会保障体系不断健全并向农村延伸。城乡居民生活水平不断提升，城镇居民可支配收入、农民人均纯收入分别达到12455元和4270元，较“十五”末分别增加6100元和1994元。五年实现55万人脱贫。

　　——这五年是周口形象改善、人气提升的五年。全市上下围绕建设“繁荣富裕、文明开放、和谐稳定新周口”的奋斗目标，解放思想，开拓创新，聚精会神搞建设，一心一意谋发展，坚持靠发展树立形象，努力营造干事创业的浓厚氛围，着力打造开放开明的发展环境，社会主义核心价值体系建设全面加强，涌现出洪战辉、王文田、李灵、陈海宏等一批道德模范和英模群体，树立了文明进步的对外形象。

　　各位代表，“十一五”的发展成就，归功于省委、省政府和市委的正确领导，得益于历届领导班子打下的良好基础，离不开市人大、市政协的监督支持，凝结着全市广大干部群众的心血和汗水。在此，我代表市人民政府，向全市广大工人、农民、知识分子、干部、驻周部队指战员、武警官兵、公安干警以及各界人士，致以崇高的敬意！向中央、省驻周单位，在周口创业发展的各位投资者和建设者以及所有关心支持周口现代化建设的同志们、朋友们，表示衷心的感谢！

　　各位代表，“十一五”时期取得的成绩来之不易，积累的经验弥足珍贵。通过实践，我们进一步深化了对科学发展观的认识，提高了贯彻落实科学发展观的能力。我们深刻体会到：一是必须坚持解放思想、改革创新、开放开明，打破思想禁锢，转变思维方式，使工作不断有新突破、实践不断有新创造、发展不断有新跨越；二是必须坚持科学发展，加快转变经济发展方式，遵循经济规律，尊重群众意愿，保持经济又好又快发展；三是必须坚持围绕经济建设中心、紧贴民心、把握和谐稳定重心的工作布局，整体推进不偏颇；四是必须坚持推进“三化”相互促进、同步发展，持续探索走好一条不以牺牲农业和粮食、生态和环境为代价的“三化”协调科学发展的路子；五是必须坚持正确处理改革发展稳定的关系，把改善民生作为三者的重要结合点，千方百计加快发展，积极稳妥推进改革，尽心尽力改善民生；六是必须坚持狠抓作风建设不放松，营造不埋怨、不争论、不折腾，和谐向上、干事创业的浓厚氛围。

　　各位代表，在肯定成绩的同时，我们也清醒地认识到，我市人口多、基础差、底子薄，部分经济指标低于全省平均水平的基本市情仍然没有发生根本改变，经济社会发展中还存在着一些困难和问题。一是加快经济发展面临诸多挑战。经济发展外部不确定因素增多，资源环境约束不断加大，区域竞争日趋激烈，保持经济平稳较快发展、缩小与全省平均水平的差距难度增大。二是经济发展还存在一些薄弱环节。经济增长方式粗放，产业层次低；工业经济总量小，主导作用不突出；投资不足，具有牵动力和支撑力的大项目少；城镇化水平低，中心城区辐射带动能力不强；农业抗灾减灾能力较弱，农业稳定增长和农民持续增收基础不牢。三是保障改善民生任务繁重。人均发展水平和人均公共服务水平低的状况没有根本改变，与人民群众过上美好生活的强烈愿望还有一定差距。事关民生的城乡基础设施落后，文化、教育、卫生等社会事业发展水平不高，历史欠账较多。四是政府工作还存在不少问题和不足。一些部门职能转变还不到位，作风不实，执行力不强；一些干部思想解放不够，服务意识淡漠，推动科学发展的能力有待进一步提高；个别干部以权谋私、奢侈浪费等现象仍然存在。对此，我们一定要高度重视，认真加以解决。

　　二、“十二五”发展总体思路和目标任务

　　“十二五”时期，是周口全面建设小康社会的关键时期，是深化改革开放、加快转变经济发展方式的攻坚时期，是实现追赶跨越发展的加速时期，是缩小发展差距的重要战略机遇期。周口已进入工业化城镇化加速推进、全面建设农业现代化的新阶段。这一时期，面临的挑战很多，但机遇大于挑战。一是政策机遇。国家实施扩大内需战略，加大支持扶持“三农”力度，统筹城乡发展，完善基础设施，加大对社会事业和改善民生的投入，有利于我们争取政策支持。二是产业转移机遇。经济全球化深入发展，国际和沿海地区产业转移步伐加快，有助于我们借助外力、提升发展层次。三是区域经济加快发展机遇。国家启动“十二五”规划、促进中部崛起，我省加快中原经济区建设，有利于我们加快“三化”进程、提高区域竞争力。四是自身基础条件机遇。经过“十一五”的发展，我们初步探索出了符合周口实际的发展思路，建立了较为完善的体制机制，形成了心无旁骛抓发展、集中精力搞建设的干事创业氛围，凝聚起加快追赶跨越的强大合力。我们必须抢抓机遇，迎难而上，坚定不移地朝着既定目标扎实迈进。

　　根据市委二届十次全会精神，“十二五”时期经济社会发展的指导思想是：深入贯彻落实科学发展观，以科学发展为主题，以加快转变经济发展方式为主线，以富民强市为中心任务，坚持“四个重在”的实践要领，遵循经济规律，尊重群众意愿，着力经济结构调整，着力改革开放，着力改善民生，促进经济又好又快发展和社会和谐稳定，为全面建设小康社会奠定坚实基础。

　　“十二五”时期的发展目标是：“一高一低、快追赶、惠民生”，经济发展速度高于全省平均水平，人口自然增长率低于全省平均水平，综合实力明显提升，经济结构明显优化，居民收入明显增加，社会建设明显加强，改革开放明显推进，不断缩小与全省平均水平差距。到2015年，力争实现“两个突破、三个提升”，即经济总量突破1900亿元；地方财政一般预算收入突破70亿元；实现城镇化率的快速提升，城镇化率达到40%以上；实现工业整体实力的快速提升，第二产业占生产总值比重达到50%左右；实现农村生产生活水平的快速提升，农村面貌发生明显变化。

　　实现上述目标任务，必须把握好五个方面，突出四项重点工作。

　　五个方面：一是以科学发展为主题，一切从实际出发，统筹各项事业、各项工作、各方力量，推动“三化同步”，实现全面协调可持续发展；二是以加快转变经济发展方式为主线，按照“高质量、优结构、可持续、惠民生”的方向，加快经济结构优化调整，在发展中转变、在转变中发展；三是以投资拉动、项目带动为抓手，充分发挥投资对经济增长的第一启动力作用，把经济、政治、社会、文化建设转化为具体项目，谋划一批事关发展全局、经济效益好、支撑能力强的重大项目，以项目带动要素聚集、带动发展提升；四是以改革开放为动力，加快重要领域和关键环节的改革，创新体制机制，增强自主创新能力，拓展对外开放的广度和深度，提供有利于科学发展的体制保障；五是以富民强市为中心任务，从人民群众的根本利益出发谋发展、促发展，激发人民群众发展的积极性，形成经济社会又好又快发展的强大合力，实现富民与强市的有机统一。

　　四项重点工作：一是着力发挥好粮食生产的重要支撑作用，全面推进农业现代化；二是着力发展好具有较强竞争力的特色主导产业，加速推进新型工业化；三是着力统筹好城乡发展，加速推进新型城镇化；四是着力保障和改善好民生，维护社会和谐稳定。

　　《周口市国民经济和社会发展第十二个五年规划纲要（草案）》及说明已印发各位代表，请予审议。

　　各位代表，“十二五”规划已经开启，我们面临的任务繁重而艰巨，肩负的使命伟大而光荣。尽管前进的道路不会平坦，但任何困难都动摇不了我们加快发展的决心和信心！我们将抓住一切机遇，积极开拓进取，求真务实，埋头苦干，努力把改革发展稳定各项工作做得更好，让周口人民过上更加幸福美好的生活！

　　三、2011年工作的主要目标和任务

　　2011年是实施“十二五”规划开局之年。做好今年的工作，对于保持经济社会发展良好势头，为“十二五”开好头、起好步具有重要意义。

　　今年政府工作的总体要求是：全面贯彻党的十七大和十七届三中、四中、五中全会精神，认真落实中央经济工作会议、省委八届十一次全会、省委经济工作会议和市委二届十次全会精神，坚持“四个重在”实践要领，抓住中原经济区建设的重大机遇，以科学发展为主题，以加快转变经济发展方式为主线，以富民强市为中心任务，以项目建设、招商引资和产业集聚区建设为抓手，以改革开放为动力，以改善民生为目的，推进“三化”协调发展，实现经济社会各项工作新突破，确保实现“十二五”发展良好开局。

　　今年经济社会发展主要预期目标是：生产总值增长10%；地方财政一般预算收入增长12%；全社会固定资产投资增长20%；社会消费品零售总额增长16%；外贸出口增长22%；实际利用外资增长22%；居民消费价格涨幅控制在4%左右；城镇居民人均可支配收入增长9%，农民人均纯收入增长9%；人口自然增长率控制在6.5％。以下；城镇新增就业7万人，城镇登记失业率控制在4.2%以内；节能减排完成省定目标。

　　围绕上述要求和目标，重点抓好以下八个方面工作：

　　（一）坚持项目带动不动摇，在增强发展后劲上实现新突破

　　今年我市在完成国家、省定投资项目的同时，筛选了100个重点项目，总投资222.5亿元。在整体推进的同时，突出抓好事关经济发展、具有重大支撑和带动作用的“双十五工程”等重点项目。

　　——突出抓好15项重点工业项目。15项重点工业项目，总投资62.4亿元，今年投资32.48亿元。

　　1.总投资11.68亿元，今年投资3亿元，开工建设沈丘金丝猴集团食品加工项目；

　　2.总投资4.67亿元，今年投资2.7亿元，开工建设鹿邑宋河酒业产业升级项目；

　　3.总投资2.6亿元，今年完成全部投资，开工并建成郸城金丹乳酸公司乳酸钠生产及L乳酸机电一体化项目；

　　4.总投资2亿元，今年投资1.5亿元，开工建设川汇区台南制衣年产160万件服装项目；

　　5.总投资1.5亿元，今年投资1.1亿元，开工建设扶沟日神服饰年加工50万件羊绒衫项目；

　　6.总投资6亿元，今年投资2亿元，继续建设商水万果园农产品深加工项目；

　　7.总投资3.5亿元，今年投资1.5亿元，继续建设川汇区长城门业“双百万工程”项目；

　　8.总投资3.5亿元，今年投资1.5亿元，继续建设项城市纺织有限公司25万锭纺织项目；

　　9.总投资2.04亿元，今年投资1亿元，继续建设开发区科信电缆碳纤维复合芯导线项目；

　　10.总投资5.4亿元，今年完成剩余投资3亿元，建成鹿邑辅仁药业年产150亿片（粒/袋）固体口服液项目；

　　11.总投资5.06亿元，今年完成剩余投资3.06亿元，建成开发区大河林业中、高密度纤维板项目；

　　12.总投资4.3亿元，今年完成剩余投资3.3亿元，建成太康万利源棉业20万锭精纺项目；

　　13.总投资4.25亿元，今年完成剩余投资3.02亿元，建成郸城晋鑫化工年产25万吨合成氨项目；

　　14.总投资2.9亿元，今年完成剩余投资1.7亿元，建成大用集团西华分公司肉鸡屠宰及饲料加工项目；

　　15.总投资3亿元，今年完成剩余投资1.5亿元，建成淮阳平康电气高压电气设备生产项目。

　　——突出抓好15项重点基础设施项目。15项重点基础设施项目，总投资59.84亿元，今年投资15.59亿元。

　　1.总投资3.6亿元，今年投资1亿元，开工建设S210永登高速至郸城段升级改造工程；

　　2.总投资2.68亿元，今年完成全部投资，开工并建成周口500千伏变电站扩建工程；

　　3.总投资1.44亿元，今年投资8000万元，开工建设周口市火车站广场及配套路网工程；

　　4.总投资1.1亿元，今年投资3000万元，开工建设周口市东新区中学项目；

　　5.总投资1.08亿元，今年投资5000万元，开工建设G311线扶沟方庄至李堂段；

　　6.总投资8900万元，今年投资5500万元，开工建设周口市八一路沙颍河桥工程；

　　7.总投资8000万元，今年投资4000万元，开工建设周口市综合客运总站项目；

　　8.总投资5800万元，今年投资2900万元，开工建设周口市第六人民医院项目；

　　9.总投资2.78亿元，今年投资1亿元，继续建设周口市体育场项目；

　　10.总投资1.15亿元，今年投资2800万元，继续建设周口市中心医院门诊楼项目；

　　11.总投资1.1亿元，今年投资5000万元，继续建设周口广播电视多功能发射塔项目；

　　12.总投资34亿元，今年完成剩余投资4亿元，建成漯阜铁路（周口段）电气化改造项目；

　　13.总投资4.77亿元，今年完成剩余投资1.26亿元，建成涡河（周口段）航运开发一期项目；

　　14.总投资3亿元，今年完成剩余投资1.3亿元，建成周口市黄淮物流农产品批发大市场项目；

　　15.总投资8760万元，今年完成剩余投资7260万元，建成周口市妇女儿童医院项目。

　　对于进入市级的重点项目，尤其是进入“双十五工程”的项目，给予优先扶持，强化环境保障。继续坚持“一个项目、一名领导、一套班子、一条龙服务”的推进机制，确保项目顺利实施。

　　（二）坚持优先发展工业不动摇，在壮大工业经济实力上实现新突破

　　突出工业主导地位，走新型工业化道路，全面提高工业经济质量效益，力争今年全市规模以上工业增加值增长15%、利润增长15%以上。

　　培育壮大支柱产业。以调整、优化和提高为方向，以研发、创新和增值为重点，做大做强三大支柱产业，促进产业链向精深加工延伸。食品加工业以发展高附加值的品牌食品为重点，扶持莲花味精、志元食品、金丝猴集团等企业扩大生产规模，加快大用邦杰西华分公司3万吨熟食、金农实业黄花菜深加工等项目建设，延伸益海粮油、五得利面粉等企业产业链条，打造农副产品精深加工产业中心。纺织服装业积极推进粤兴纺织高档优质纱、德生服饰高档面料印染、宏星制衣等项目建设，引导鑫达、中方、昌茂等企业扩大生产规模，发挥企业规模效益，提升纺织服装生产规模和产业竞争力。医药化工业鼓励引导龙都药业、帅克药业、四方药业等企业，进一步增强自主研发能力，加快新型药品研制。以所望化妆、金丹乳酸等企业为龙头，大力培育日用化工产业。

　　加快发展新兴产业。以科信电缆、阳光电缆、久通电缆为重点，大力发展通信电缆产业，尽快形成在国内有影响的电缆生产基地。充分利用省政府确定我市为生物科技生产基地机遇，以辅仁药业、远东农药和其它生物科技企业为重点，加大科技投入，推动生物科技产业健康快速发展。以银丰塑料、龙源纸业、迪冉纸业为依托，大力发展彩印包装产业。

　　扶优扶强骨干企业。支持30户重点企业和10户高成长性企业与国内外500强、同行业100强联姻结亲，吸纳优势资源，实现规模扩张。鼓励益海粮油、财鑫集团、辅仁集团等行业龙头企业兼并困难企业，推进关联产业、上下游企业融合重组，争取新增一批销售收入超10亿元的企业。

　　全力抓好产业集聚区建设。全面启动港口及综合物流集聚区建设，进一步加快11个产业集聚区基础设施建设。加快产业集聚区道路、供排水等基础设施与区外设施互通对接，同步规划建设生活服务设施，为人口有序流动和企业后勤保障创造更加有利条件。在加快集聚区现有项目建设的同时，着力引进一批对特色主导产业带动作用强的龙头企业，促进中小企业集聚发展，提升产业集聚效应。今年每个产业集聚区都要有2～3个亿元以上项目落地，11个产业集聚区要完成各类投资180亿元以上。

　　深化企业服务。密切关注宏观经济运行环境变化，加强对重点行业、骨干企业和主要产品的市场监测分析，努力化解经济运行中的不利因素。搞好煤、电、油、运等生产要素的调度，确保企业正常生产经营。建立完善政策导向、市场开拓、融资服务、困难企业帮扶等长效服务机制，市、县联合抓好100家重点企业，实施年度动态管理。加大金融资本与产业资本对接力度，搭建政府、金融机构、企业合作平台，形成多方共赢的局面。积极扩大融资规模，提升资本市场融资能力，满足企业资金需求。支持金丹公司等企业上市，鼓励企业利用多层次资本市场体系融资，逐步缓解企业融资难题。

　　（三）坚持农业基础地位不动摇，在“两区一体系”建设上实现新突破

　　坚持把解决好“三农”问题作为各项工作的重中之重，立足我市实际，重点抓好粮食生产核心示范区、现代农业示范区、农业服务体系建设，全面建设农业现代化，为加速推进工业化、城镇化提供坚实基础。

　　抓好粮食生产核心示范区建设。围绕“十二五”末全市粮食生产能力达到165亿斤目标，大力开展农业基础设施建设。从今年起，每年建设20万亩高产稳产田，改造26万亩中低产田。抓住国家加大水利投资机遇，多渠道增加投入，大兴农田水利基本建设。组织实施好小型农田水利重点县建设，继续搞好槐店灌区续建配套与节水改造项目，完善农田灌排体系；完成重点平原洼地利用外资项目和白沟河、新蔡河、老涡河等中小河流治理项目，夯实农业稳产增产基础。开展高产创建活动，在抓好全市38个小麦、28个玉米万亩高产创建示范区的基础上，每个县市建设3个以乡镇为单位整建制粮食高产示范区，辐射带动全市粮食均衡增产。认真落实强农惠农政策，保护和调动农民种粮积极性，力争今年粮食总产突破145亿斤。

　　抓好现代农业示范区建设。坚持用现代物质条件装备农业、用现代科学技术改造农业、用现代产业体系提升农业、用现代经营形式推进农业。积极推进农业基础设施建设。加快农业机械设备和农机技术推广，新增各类大中型农业机械2000台、农机总动力30万千瓦。继续实施农村电网升级改造，不断改善农村电网现状。大力推进农业科技创新和应用，进一步完善农业科技推广服务模式，继续开展服务粮食生产科技行动。大力实施阳光工程等项目，建立一批实用人才培训基地，加快提升农业劳动者的科技文化素质。着力调整农村产业结构，大力发展农产品加工业和劳务经济。积极探索建立农产品精深加工集聚区，实现农产品转化增值。着力调整种植业结构，努力提高种植效益。在扶沟办好河南省第四届蔬菜双交会，提高周口蔬菜知名度。强力推进畜牧业发展。力争年出栏家禽突破1亿只、出栏生猪达到600万头。大力推进农业产业化经营。继续实施“50家龙头企业培育工程”，进一步培强做大龙头企业。实施“50个农业产业化生产基地建设工程”，保障企业原料供应，促进农民持续增收。大力发展各种类型的农民专业合作社，力争达到3500家。开展示范区试点工作。借鉴西华龙池头村万亩高产示范方建设模式，引导各县市区强化资源合理配置，整合涉农资金，用于现代农业示范区建设。各县市区结合自身农业基础和产业优势，认真谋划建立各具特色的现代农业示范区，形成“一县一品”、“一县一特色”，以点带面，全面推进现代农业示范区建设。

　　抓好农业服务体系建设。加强农业科技推广体系建设，力争年内在全市建立健全乡镇或区域性农业技术推广、动植物疫病防控等公共服务机构，努力提升关键生产环节的专业化统一技术服务能力。加强农产品质量检测和基层动物防疫体系建设，建立健全市、县两级农产品质量检测监管机构，以及乡镇动物疫病防检中心。加快农业信息体系建设，着力构建以市、县农业信息部门为龙头，乡镇信息服务站为枢纽，农业产业化重点龙头企业、农产品批发市场、种养大户和农村信息服务站为节点的工作体系，不断提高信息服务“三农”水平。

　　抓好新农村建设。进一步加大新农村建设投入力度。继续推进新农村“111”工程，抓好10个示范乡镇和100个示范村建设。因地制宜，突出重点，统筹做好扶贫开发、饮水安全、农村道路建设、林业生态建设、村容村貌整治等各项基础性工作。实施150个贫困村整体推进扶贫，帮助8万农村贫困人口脱贫。解决60万农村居民的安全饮水问题。继续实施“乡乡联、县县畅”工程，改造农村公路400公里、危桥26座。完成省政府下达的新增农村户用沼气和大中型养殖场沼气建设任务。

　　（四）坚持推进城镇化进程不动摇，在统筹城乡协调发展上实现新突破

　　实施城乡建设三年大提升行动计划，加快形成中心城区、县城、重点小城镇和新型农村社区四个层次的城镇化体系。强化中心城市地位，提升城市综合经济实力，增强城市对区域经济的聚集能力和辐射能力。大力发展县域经济，实现城乡统筹发展。

　　加快中心城市发展。按照“综合实力明显增强、城市形象明显改善、群众生活水平明显提升”的要求，在培育产业、完善功能、加强管理、提升品位上下工夫，继续修编完善城市总体规划，努力推进中心城市科学发展。大力培育城市产业。认真研究中心城区产业经济发展规律，制定鼓励支持中心城区二三产业发展政策，落实好《关于加快周口中心城区发展服务业的若干意见》，从服务、环境、政策、机制等各个方面入手，全力扶持中心城区二三产业发展壮大。积极推进“三区”协调联动发展。做大做强开发区。按照打造中心城区工业基地、集聚城市产业要求，进一步加大招商引资和产业集聚力度。加快利杰玻璃、奇美电子等项目建设，确保年内建成投产。启动企业服务中心项目建设，完成高新科技园区现代标准化厂房建设任务，为企业入驻提供条件。加快建设东新区。以区划调整为契机，逐步完善基础设施，扎实推进移动公司、河南建业等单位项目建设，争取尽快出形象、聚人气、显亮点。年内开工建设文昌大道慢车道延伸线、师院路和流沙河改造工程，建成平安路、光明路等5条道路。改造提升老城区。加大老城区芙蓉路、育新街等道路改造力度。完成36万平方米棚户区改造任务。打通部分街道断头路，亮化、绿化、美化小街小巷，努力改善市民居住环境。加强城市供水、供电、通讯、燃气、公厕、公交等基础设施建设，健全教育、文化、医疗卫生等公共设施，全面提升中心城市生产生活要素保障能力。加强城市管理。继续开展“两违”综合整治活动。理顺城市管理体制，创新城市管理机制，将城市精细化管理工作纳入目标考核，严格考评，努力创建宜居生态城市。加快中心城区社区建设，加强小区物业管理和小街小巷环境治理，不断提高城市管理水平。

　　加快县城和小城镇建设。加强城镇交通、通讯、供水、供气、供电和污水、垃圾处理等基础设施建设，完善城镇功能。坚持把县城和小城镇建设与市场建设、产业集聚发展结合起来，增强城镇综合经济实力和辐射力，引导人口向城镇发展、产业向集聚区集中，以人口和产业集聚带动县城规模扩大和中心城镇发展，形成优势互补、各具特色的中小城市格局。

　　积极稳妥推进新型农村社区建设。坚持规划先行、就业为本、群众自愿、量力而行的原则，加强政策引导，因地制宜、分类推进农村社区建设。加快城市建成区内城中村改造步伐，实施中心城区沙北滨河棚户区改造工程，启动60个农村社区建设试点工作。产业集聚区、城市新区和中心城市近郊区的村庄，率先建设新型农村社区。

　　大力发展县域经济。引导县市立足自身特点和优势，依托本地资源条件和现有基础，采取加快工业发展、扩大招商引资、推进项目建设、壮大民营经济、创新体制机制五项举措，因地制宜，发挥优势，建设各具特色的产业集群，培育县域经济支柱产业和龙头企业，形成特色鲜明的县域经济发展格局，不断提升各县市在全省整体排名中的位次。从政策、信息、资金、人才等各个方面支持县域经济发展，特别是在工业发展和产业集聚区建设上，帮助各县市解决发展规划、用地指标、融资平台建设等方面的问题。

　　（五）坚持加快转变经济发展方式不动摇，在促进经济又好又快发展上实现新突破

　　立足资源、区位优势，把加快结构调整作为转变经济发展方式的主攻方向，在发展中促转变，在转变中促发展。

　　提高自主创新能力。继续加强与省科技厅的工作会商，加大与高校、科研院所的合作力度。加快企业技术改造步伐，切实抓好亚华玻璃技术改造等80个技改项目，推动产业、产品结构转型升级。进一步加强企业研发中心、重点实验室等创新平台建设，力争创建省级企业研发中心5个、市级企业研发中心20个。加快省鞋制品产品质量监督检验中心、国家河南周口特种设备能效测试中心等建设，强化质量监管。实施名牌带动战略，力争年内新增河南省名牌产品5个。深入开展质量兴市活动，创建国家4A级标准化良好行为企业1个、国家级服务标准化示范单位1个、国家级农业标准化示范区1个，培育市长质量奖企业5家。

　　扎实推进节能减排和生态环境建设。大力发展循环经济，加大对氨氮排放量较大行业的治理力度，深入开展大气污染防治。继续实施八大节能工程，淘汰高耗能设备。实行最严格的水资源管理制度，建立节水型社会。加快城市污水处理厂升级改造和管网配套建设，确保平均运行负荷达到80%以上。积极推进林业生态市和国家现代林业示范市建设，完成植树2800万株任务。每个县市区抓好1个生态乡镇和5个生态村建设。深入开展“创建国土资源节约集约模范县（市）”活动，实行最严格的耕地保护制度和土地节约制度，确保国土资源有效保护、有序开发。

　　加快发展服务业。改造提升餐饮、商贸等传统服务业，规范发展商务、金融、商业保险、专利代理、会计、评估、咨询等现代服务业。积极发展教育培训、保健健身、家政代理等新兴服务业。进一步抓好重点物流项目，不断提高我市现代物流业水平，打造豫东南商贸流通产业中心。继续实施“万村千乡”市场工程，落实“家电下乡”、“家电以旧换新”等优惠政策，活跃农村商贸市场，扩大农村消费。

　　（六）坚持建设文化强市不动摇，在文化旅游融合发展上实现新突破

　　充分发挥周口文化底蕴深厚、地域特色鲜明的优势，大力弘扬伏羲文化和老子道家文化，认真谋划和推动文化事业和文化旅游产业发展，促进文化与旅游的融合。

　　加快发展文化事业。全面落实文化惠民工程，继续实施农家书屋和文化信息资源共享、农村地面数字电视“村村通”等工程，完成乡镇综合文化站达标建设，提升基层文化设施水平。进一步打造公益性文化品牌“周末一元剧场”。加强文物和非物质文化遗产保护与开发利用，争取将第二届河南省非物质文化遗产展演扩大到中部六省。加大监管力度，繁荣文化市场。加快完善市文化艺术中心内部功能，年内投入使用。

　　大力发展文化产业。认真落实文化产业扶持政策，大力发展网络服务、广告会展、文化创意等新兴文化产业，支持文化娱乐、艺术培训等社会文化产业发展。整合书画经营、杂技培训与演出等文化资源，发展特色文化产业集群，建设周口关帝庙文化产品经营示范区。推进淮阳文化改革发展试验区建设，举办第五届龙湖荷花节。

　　着力发展旅游业。夯实羲皇故都国家5A级景区创建基础，完成淮阳国家旅游标准化示范县创建任务。切实抓好老子故里、伏羲太昊陵2个国家4A级景区，关帝庙、吉鸿昌将军纪念馆2个国家3A级景区，以及女娲城、袁世凯故居、中原民俗园景区的基础设施建设和管理，不断提升景区内涵和品位。完善服务功能，推动旅游业转型升级。

　　（七）坚持改革开放不动摇，在破解制约发展瓶颈和难题上实现新突破

　　认真研究解决经济社会发展中的突出矛盾和深层次问题，深入推进重点领域和关键环节的改革创新。

　　全面深化各项改革。深化行政审批制度改革和投资体制改革，完成机关直属企业脱钩改制，推进政府投资评审、政府采购、国有资产管理等财政体制改革。支持税务部门征管改革。继续深化农村综合改革，积极推进村级公益事业“一事一议”工作。加快县、乡土地流转服务平台建设，2/3的乡镇建立农村土地流转服务大厅，力争全市规范性土地流转面积达到150万亩。建立健全林业经济合作组织和林木产权交易平台，完成集体林权制度改革任务。深化医药卫生体制改革，鼓励民营资本创办非营利性医院，建立健全覆盖全市城乡居民的基本医疗卫生制度。积极稳妥推进商贸、物资、交通、文化、教育、水利等行业改革。支持金融部门改革，鼓励金融部门向产业集聚区、农村延伸服务机构。

　　深入开展大招商活动。利用大招商活动的综合、集聚效应和小分队招商、驻地招商的灵活性，提高招商引资针对性和实效性，推动与沿海经济发达地区战略合作，积极承接产业转移，加快引进一批大项目。依托现有外商投资企业，推动以商招商，引进外商投资开发新项目或鼓励企业增资扩股。注重招商引资质量，重点选择有一定产业基础、高成长性的行业，积极引进一批高附加值、高技术含量、高产业关联度的项目。实施“招行引资”战略，争取全国性股份制商业银行进驻周口，依法开展业务。加快科技、教育、文化、卫生、体育、水利等领域开放步伐，吸引更多境内外资金进入高技术产业、现代服务业和现代农业，参与城镇化和农村基础设施建设，形成宽领域对外开放格局。

　　积极扩大外贸出口。紧紧抓住省政府确定我市为“河南省制鞋产业出口基地”机遇，以大招商、大开放、促大发展活动为契机，大力引进投资规模大、出口带动能力强的项目落户我市，着力培育一批出口产业基地和产业集群，增强出口后劲。认真落实重点进出口企业联系制度，帮助解决企业实际困难和问题，保持进出口持续稳定增长。

　　（八）坚持改善民生不动摇，在和谐周口建设上实现新突破

　　进一步加大投入，继续实施好“十项民生工程”，扎扎实实为群众办实事、解难事。

　　千方百计扩大就业再就业。实施更加积极的就业政策，多渠道开发就业岗位，扶持自主创业和自谋职业。加强就业技能培训、就业信息服务和就业援助，重点解决高校毕业生、农村劳动力和城镇就业困难人员就业问题。完善城乡公共就业服务体系，建立规范的人力资源市场，为劳动者提供优质高效的就业服务，力争下岗失业人员再就业3万人，其中就业困难人员再就业1万人，新增农村劳动力转移就业10万人。

　　逐步完善社会保障体系。健全企业退休人员基本养老金、城乡居民低保、农村五保户供养、优抚对象和孤儿基本生活最低养育标准正常调整机制。继续提高企业退休人员基本养老金水平，在上年人均基础上再提高10%左右。总结推广扶沟、项城新农保试点工作经验，逐步扩大新农保试点范围。切实解决好失地农民生活保障问题。完善城镇职工、居民医疗保险制度，全面实现城镇基本医疗保险即时结算和医疗保险、工伤保险市级统筹。提高城镇居民医保、新农合财政补助标准，全面实行新农合就地直补和居民医保门诊统筹，扩大新农合门诊统筹试点。完善城乡社会救助体系，建立健全临时救助机制。大力发展残疾人事业、社会福利和慈善事业。落实“菜篮子”市长负责制，抓好新一轮“菜篮子”工程建设。开工建设各类保障性住房8470套49万平方米，逐步缓解困难群体住房难问题。

　　积极发展各项社会事业。进一步组织好文化、科技、法律、卫生“四下乡”和科技、文体、法律、卫生“四进社区”活动，力争创建5个国家科普示范县（市），提高全民科学文化素质。认真落实全市教育工作会议精神及《周口市中长期教育改革和发展规划》，加大教育投入，重点解决好农村中小学布局调整、中心城区“大班额”和入学难问题。优化资源配置，推进学前教育和义务教育均衡发展。加强市职业教育园区、县职业教育中心和教师队伍建设，全面提高教学水平。积极实施《周口市中长期人才发展规划纲要》，加强人才队伍建设。继续抓好县级医院、乡镇卫生院、标准化村卫生室和城市社区卫生服务中心等项目建设，健全城乡医疗卫生服务体系。启动全市精神卫生防治体系和卫生执法监督体系基础设施建设项目。实施国家“健康快车”周口行白内障复明工程。广泛开展全民健身活动，促进群众体育和竞技体育共同发展。创新计生家庭奖励机制，稳定低生育水平，确保人口自然增长率低于全省平均水平。继续做好第六次全国人口普查工作。完成第二轮续志任务。

　　努力提高全民文明素质。深入开展公民素质教育，扎实推进社会主义核心价值体系建设，塑造诚信周口、文明周口、礼仪周口的良好形象。开展创建文明县城、文明行业、文明单位、文明社区活动，不断提高城市文明程度。积极创建卫生城市。大力推进“清洁家园行动”和文明村镇、文明集市、文明卫生户创评活动，继续深化文明单位结对帮扶和城乡共建工作，着力提升农村精神文明创建水平。深入开展“扫黄打非”，加强未成年人思想道德教育、校外活动场所建设，进一步净化社会文化环境，不断提高未成年人思想道德建设水平。积极开展群众性精神文明创建和志愿服务活动，形成扶正祛邪、惩恶扬善的良好社会风气。

　　加强和创新社会管理。扎实推进平安周口建设，完善社会治安防控体系，依法打击各类违法犯罪活动。加强和改进新形势下群众工作，深入推进社会矛盾化解、社会管理创新、公正廉洁执法三项重点工作，最大限度减少不和谐因素。建立完善矛盾调处和社会利益协调机制，从源头上预防和减少矛盾发生。加强法律援助，依法保障公民特别是妇女、未成年人和残疾人的合法权益。继续开展“安全周口”创建活动，加强烟花爆竹、道路交通、消防、危险化学品等重点领域和行业的监管，有效防范各类事故发生。大力整顿和规范市场经济秩序，切实加强食品药品和食盐安全监管，保障人民群众身体健康。健全应急管理体系，提高预防和处置突发公共事件能力，维护社会大局稳定。

　　大力加强民族宗教工作，促进各民族共同团结进步、共同繁荣发展。深入开展拥军优属和军民共建活动，关心、支持驻周解放军、武警消防部队和民兵预备役建设，进一步密切军政军民关系。充分发挥各民主党派、工商联、无党派人士的参政议政作用，积极支持工会、共青团、妇联等群众团体开展工作。继续做好审计、统计、海关、物价、气象、地震、人防、外事、侨务等工作，促进社会事业全面发展。

　　四、建设人民满意的政府

　　各位代表，做好今年的政府工作，战胜各种困难和挑战，需要政府有更大的作为，需要政府工作人员付出更大的努力。我们将继续推进政府管理创新，按照科学化、规范化、制度化的要求，完善规则，建章立制，以领导方式转变加快发展方式转变，着力提高推动科学发展的能力，努力建设人民满意的政府。

　　建设学习型政府。坚持学习与思考并重、学习与工作一体，采取行政学院培训等多种形式，有计划、有针对性地开展公务员培训工作，大力营造重视学习、勤于学习、善于学习的良好氛围，不断增强驾驭市场经济和解决实际问题的能力。密切关注形势变化，准确把握政策导向，加强对外沟通联系，深入掌握基层动态，不断更新知识结构，切实将各级的利好政策、各地的经验做法、各方的先进理念与我市的实际情况有机结合起来，转化为推动发展的思路、举措和行动，使决策更加科学合理、措施更加务实有效。

　　建设服务型政府。树立正确的政绩观，始终把为民服务作为执政之本、工作之要，增强服务意识，扎扎实实为基层、企业和群众办实事、解难题。进一步加强行政服务中心建设，不断创新服务方式，努力提高服务水平。继续广泛开展民主评议市直单位及其重点科室（二级机构）活动，切实解决办事效率不高、服务质量不优和门难进、脸难看、事难办等问题。多渠道了解群众愿望，倾听群众呼声，关注群众疾苦，解决好人民群众最关心、最直接、最现实的利益问题。尊重劳动、尊重知识、尊重人才、尊重创造，积极为人民群众干事创业、发挥特长搭建平台，为实现人的全面发展创造良好环境。

　　建设效能型政府。继续发扬严、细、深、实、正的良好作风，强化运作和执行能力。改革政府管理体制，理顺政府部门分工和权责关系，强化协调联动，形成推动工作的整体合力。进一步减少和规范行政审批事项，精减会议文件，加强和改进“一站式”服务。深化政务公开，强化效能监察，加快电子政务和行政效能电子监察系统建设。创新政府管理方式，定制度、定标准、定措施、定职责，将政府工作的主要任务层层分解，责任到人，强化绩效评估，严格兑现奖惩。各级政府及其工作人员都要以对工作高度负责的精神，以时不我待、只争朝夕的状态，集中精力解难题，争分夺秒抓落实，扎扎实实见成效，确保各项目标任务顺利完成。

　　建设法治型政府。坚持把依法行政贯穿于政府工作的全过程，全面启动“六五”普法工作，认真贯彻落实《行政许可法》和《行政处罚法》，严格依照法定权限和程序行使权力，履行职责，接受监督。加强对执法部门的监管，严厉查处执法不公、越权执法、执法犯法等行为。积极推进政务公开，进一步完善重大事项公示制度和听证制度，保证人民群众依法行使知情权、参与权和监督权，推进行政决策的科学化、民主化。坚持向人大及其常委会报告工作和向政协通报工作制度，自觉接受市人大及其常委会的法律监督、工作监督，主动接受市政协、各民主党派、工商联、党外人士和各社会团体的民主监督，真诚接受广大人民群众和新闻媒体的监督，广开参政议政渠道，齐心协力推进社会主义现代化建设。

　　建设廉洁型政府。全面落实党风廉政建设责任制，更加注重标本兼治、惩防并举，更加注重完善制度、规范权力，严格执行《廉政准则》，加强反腐倡廉教育，筑牢拒腐防变的思想道德底线。坚持用制度管权、靠制度管人、按制度办事，强化对行政审批、招投标、土地出让、政府采购、国有资产产权交易等重点领域和关键环节的监管。加强领导干部任期经济责任审计和政府投资建设项目审计工作，推进审计关口前移，做到事前预防、事中管控。坚持厉行节约，反对铺张浪费，努力降低行政成本。严厉查处不作为、乱作为和违法违纪行为，切实在群众中树立求真务实、清正廉洁的良好形象。

　　各位代表，蓝图已经绘就，事业任重道远。立足新起点，再创新优势，实现新跨越，是全市人民的共同期待，是我们肩负的神圣使命。让我们携起手来，在市委的坚强领导下，万众一心，埋头苦干，克难攻坚，奋发图强，为建设繁荣富裕、文明开放、和谐稳定的新周口而努力奋斗！
